# Application of Geographic Information Systems to Address Chronic Disease Priorities: Experiences in State and Local Health Departments

**DOI:** 10.5888/pcd16.180674

**Published:** 2019-05-23

**Authors:** Ian Brissette, Michele Casper, Sara L. Huston, Melita Jordan, Becky Karns, Christopher Kippes, Michael R. Kramer, James M. Peacock, Adam S. Vaughan

**Affiliations:** 1 Bureau of Chronic Disease Evaluation and Research, New York State Department of Health, Albany, New York; 2 Division for Heart Disease and Stroke Prevention, Centers for Disease Control and Prevention, Atlanta, Georgia; 3 Maine Center for Disease Control and Prevention, Augusta, Maine, and University of Southern Maine, Portland, Maine; 4 Division of Community Health Services, New Jersey Department of Health, Trenton, New Jersey; 5 Epidemiology, Surveillance, and Informatics Services, Cuyahoga County Board of Health, Parma, Ohio; 6 Department of Epidemiology, Rollins School of Public Health, Emory University, Atlanta, Georgia; 7 Cardiovascular Health Unit, Minnesota Department of Health, St. Paul, Minnesota

**Summary**

**What is already known about this topic?**

Health departments are keenly aware of the importance of local-level data to effectively and efficiently reduce the burden of chronic disease. We asked 4 state and local health departments about their experiences using GIS to address chronic disease priorities.

**What is added by this report?**

These responses reveal the extent to which maps and spatial analyses help to 1) document the geographic patterns of chronic disease, 2) inform resource allocation and policy, 3) develop culturally competent programs, and 4) assist with program planning, monitoring, and evaluation.

**What are the implications for public health practice?**

The continued and enhanced application of GIS to chronic disease surveillance, prevention, and treatment priorities can provide valuable benefits to both health departments and the communities they serve.

## Introduction

Health department staffs are keenly aware of the importance of local-level data to effectively and efficiently reduce the burden of chronic disease (1). These local data — including data on disease burden, demographic factors, socio-environmental conditions, risk factors, and health care facilities — may be generated, analyzed, and mapped using geographic information systems (GIS), providing health department leadership and staff members with valuable information. This application of GIS (and the underlying capacity of health departments to perform GIS work) allows health departments to better incorporate chronic disease prevention activities into the places where people live, work, and play (1–3).

Given the value of applied GIS, we invited staff members from 1 local and 3 state health departments to describe their use of GIS to address chronic disease priorities. These health departments previously participated in the Building GIS Capacity for Chronic Disease Surveillance training, which is provided through a collaboration of the Centers for Disease Control and Prevention (CDC), the National Association of Chronic Disease Directors, and the Children’s Environmental Health Initiative (CEHI) (currently at Rice University) (2). The prompts given to the health departments were:

1. Please provide a brief description of how your health department currently uses GIS to address chronic disease priorities.
2. Please describe the benefits and challenges of using GIS to address the chronic disease priorities in your health department.
3. Please discuss how the use of GIS has enhanced your health department’s ability to perform one or more of the Public Health Foundation’s Core Competencies for Public Health Professionals (4) or the CDC/Council of State and Territorial Epidemiologists Applied Epidemiology Competencies (5). These competencies provide health departments with a definition of applied epidemiology and provide a framework within which to develop a public health workforce and to enhance the health of their communities.

The health departments’ answers to these prompts reveal the extent to which “Where” has become a key component in their chronic disease work. These health departments have extended basic GIS skills and maps into a more robust GIS infrastructure that supports the integration of GIS into many facets of their chronic disease programs. Critically, these health departments describe the value of using maps and spatial analyses to communicate the burden of disease; to inform decisions about resource allocation and policy, and to determine priority communities for intervention efforts; to develop culturally competent programs; and to assist with program planning, monitoring, and evaluation. Furthermore, health departments have experienced the benefits of using maps to communicate with diverse audiences, both within the health department and to partners, policy makers, and the public. Communicating disparities in the geographic patterns of chronic disease to the public enhances and improves community engagement. Finally, the health departments’ responses reveal that GIS is an important tool to support internal cross-disciplinary workgroups within an agency, to create a stronger workforce for public health programming, and to further each agency’s core mission for chronic disease prevention and management. As demonstrated in the following examples, GIS facilitates additional intersections across many of the public health and applied epidemiology core competencies, multiplying the benefits to the health departments and the communities they serve.

### Cuyahoga County, Ohio, Board of Health

#### Question 1: How is your health department using GIS to address chronic disease priorities?

The Cuyahoga County Board of Health (CCBH) participated in CDC’s Building GIS Capacity for Chronic Disease Surveillance training in 2014. Since that time, CCBH continues to expand and improve its GIS capacity (6). Our agency works in collaboration with the Cuyahoga County Planning Commission on a CDC-funded grant called Creating Healthy Communities (CHC) (7), administered by the Ohio Department of Health. CHC aims to reduce leading causes of death by increasing access to healthy foods, active living, and healthy eating; reducing smoking; and reducing childhood obesity. One outcome of this partnership was the creation of a map depicting both supermarket access and chronic disease health indicators, which was used to highlight food deserts and to examine the potential impact of future stores on neighborhood health.

**Figure 1:**
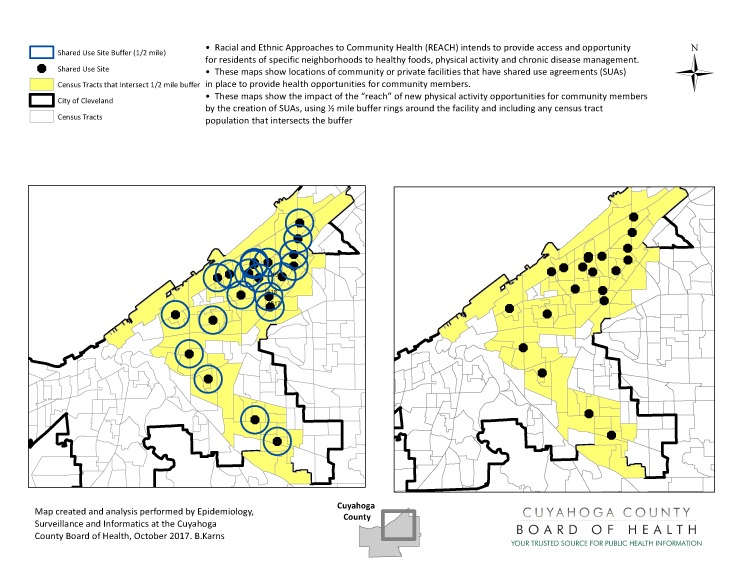
GIS map generated by Cuyahoga County Health Department to map locations of community facilities with shared-use agreements for chronic disease self-management workshops and active living activities and populations within a half mile of those facilities.

CCBH uses GIS in grant applications and reporting. CCBH has been funded for CDC’s Racial and Ethnic Approaches to Community Health program (REACH) (8). In response to the project’s focus on enhancing access to healthy foods, physical activity, and chronic disease management in communities with the greatest need, we produced a map showing locations of community facilities with shared-use agreements for chronic disease self-management workshops and active living activities (Figure 1). The maps also indicated populations living near those facilities, defined by using half-mile buffer rings around those facilities. This map then allowed us to identify nearby populations that could potentially gain access to these facilities and programs. Agreements with these facilities increased access to resources for an estimated 140,838 people who previously did not have access.

Our agency also produces cancer surveillance reports. In these reports, we analyze data for 24 types of cancer at the community level. Documenting the geographic distribution and burden of cancer helps prevention and control specialists make informed decisions and provides community-specific resources and education (9).

The Ohio Department of Health’s Healthy Homes Lead Poisoning Prevention Program provides data to CCBH to monitor blood lead levels in children. We publish annual maps of blood lead surveillance data by neighborhood and municipality (10).

CCBH provides data to a local open-data platform, Health Data Matters, where users can create custom maps of health indicators (such as chronic disease and childhood lead poisoning) (11). In addition, local communities in our jurisdiction use our GIS products in their master plans to explore the health of their community, including chronic disease death rates, life expectancy, and access to grocery stores (12).

#### Question 2: What are the benefits and challenges of using GIS to address the chronic disease priorities in your health department?

CCBH confronts some challenges when using GIS to address chronic disease: creating standardized geographic databases for consistency throughout the agency, maintaining the best and most current geographic boundary data, the expense of GIS software, time and resource allocation for staff training, and finding best practices around GIS and chronic disease mapping. Despite these challenges, our agency has formally considered GIS-based activities to be an agency-wide asset and priority by identifying GIS in the agency strategic plan, forming an agency GIS workgroup, creating a map library, and implementing a formal electronic request process. Maps are also an invaluable way to help tell a story quickly to many audiences and to advocate for specific interventions.

#### Question 3: How is your health department using GIS to enhance the Core Competencies for Public Health?

Analytical/assessment skills: CCBH recognizes the importance of applying GIS analytical skills, with staff members continuing to sharpen their skills. CCBH now has staff members, including data analysts, field staff, information specialists, and supervisors, who are part of an internal workgroup providing GIS services to the agency. The goal of the workgroup is to establish an agency-wide, location-based approach to data collection and reporting. Members of the workgroup share knowledge and skills to deepen current GIS capacity and encourage open participation from all staff members interested in broadening their GIS work.

Policy development/program planning skills: CCBH staff members use GIS for program planning and communication to advocate for interventions to community partners, stakeholders, and health care systems. The CCBH staff is often invited to speak on areas of need and uses GIS to support how communities could benefit from various interventions (eg, a new labor and delivery hospital).

Cultural competency skills: Our agency uses GIS to document geographic patterns of social determinants of health including redlining, poverty, and education, along with their geographic overlap with health outcomes, particularly on chronic diseases and infant mortality. For example, GIS was used to identify target census tracts for the REACH grant. CCBH then helped identify residents in those neighborhoods to become community health ambassadors to provide context and develop messaging for their own unique neighborhoods (13).

As in many places, in Cuyahoga County and the City of Cleveland poor health outcomes and social determinants of health exhibit similar geographic patterns. The connections between patterns of poverty, race, and child deaths have been illustrated in our annual Child Fatality Review report (14).

### Maine Center for Disease Control and Prevention

#### Question 1: How is your health department using GIS to address chronic disease priorities?

**Figure 2:**
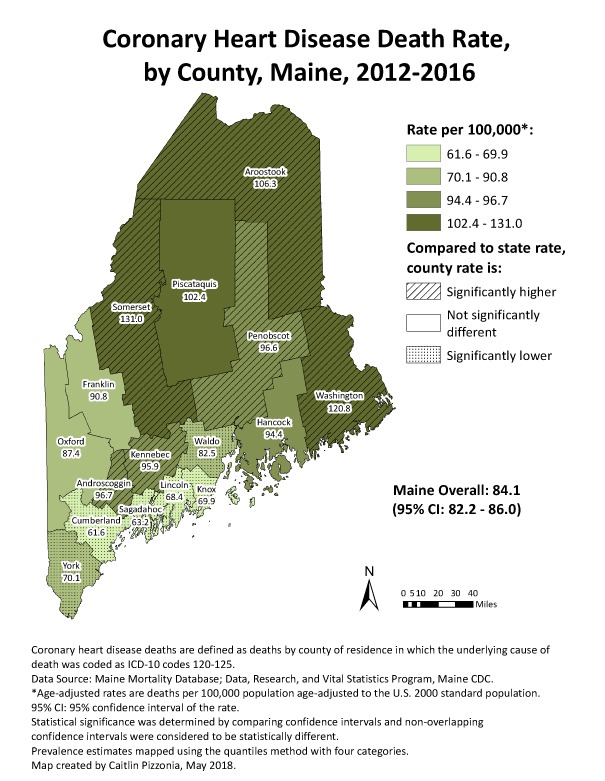
GIS map generated by the Maine Center for Disease Control and Prevention to show distribution of coronary heart disease death rates by county. Abbreviation: CI, confidence interval.

Since participating in CDC’s Building GIS Capacity for Chronic Disease Surveillance training in 2011 (15), chronic disease epidemiologists and program staff members of the Maine Center for Disease Control and Prevention have incorporated GIS into routine business across all chronic disease programs. This process began slowly with a few basic maps. Now, as a standard part of epidemiologic work in our chronic disease programs, we have systematized map-making for local chronic disease measures and surveillance indicators. Maps can also show intervention sites, local policies, health care resources, or other related information. These maps are created using standard ArcGIS templates (Esri), and are incorporated into routine public and internal products such as the Maine Cancer Registry’s Annual Report (16) and the State Public Health Actions (1305 cooperative agreement) program’s standard epidemiology figures (Figure 2) (17). Our standard map templates have evolved and now include the state rates and 95% confidence intervals and identify counties with significantly higher or lower rates than the state overall. These maps are used to identify areas of the state with high burden, need, and opportunities for intervention.

We have also begun creating interactive mapping tools using Instant Atlas (Esri) and, more recently, ArcGIS Online. We have 3 Instant Atlas products publicly available for our Maine Integrated Youth Health Survey (18), the State Public Health Actions (1305 cooperative agreement) program (17), and our Division of Disease Prevention Key Surveillance Indicators (covering chronic disease, maternal and child health, and injury) (19). These products allow users to choose the indicators and years of data, toggle between county and public health district, manipulate map characteristics, and more. We are also developing an ArcGIS Online WebApp for programmatic decision-making around blood pressure medication adherence. This product includes county-level blood pressure medication adherence and blood pressure control rates, locations of pharmacies and health care practices (including those already involved in health department–led interventions), and demographic data, such as population density. The WebApp allows the program staff to use customized maps to make programmatic decisions, such as which pharmacies or practices may be high priorities for programmatic involvement.

#### Question 2: What are the benefits and challenges of using GIS to address the chronic disease priorities in your health department?

The biggest benefit of GIS is that almost everyone loves maps, and maps are a great communication tool. Maps make very clear the areas of the state with greatest burden and need. Well-designed maps translate complex data into understandable information for public health action in a way that few other tools do. GIS products also promote discussion and collaboration among any group of people and lead to more questions and curiosity about public health issues. GIS products also help communicate to health department leadership the value of surveillance and epidemiology.

Our major challenge has been developing and retaining staff GIS expertise. Even today, not all newly minted MPH epidemiologists have GIS skills. The CEHI trainings and materials have helped us enormously with training new epidemiologists (2). Our approach has been to train all our chronic disease epidemiologists in basic GIS skills to create quality maps using our standard templates. We also have several epidemiologists with advanced GIS skills who lead development of more innovative GIS products. Developing a relationship with our Maine Office of GIS has also helped us a great deal, particularly as we move into the world of ArcGIS Online.

Another challenge is the limitation of static maps. Our Maine asthma disparities map, for instance, shows the most recent county-level asthma emergency department and hospitalization rates along with asthma program intervention sites, showing alignment between burden and intervention coverage areas. However, rates and programs change over time and these changes are extremely difficult to show clearly on a static map. As described above, we are developing interactive maps to overcome this limitation.

#### Question 3: How is your health department using GIS to enhance the Core Competencies for Public Health?

Analytical/assessment skills: Incorporating GIS into our routine work has enhanced our surveillance and epidemiology work by ensuring we are always asking and answering the critical question of “Where?” Mapping and spatial analysis have been key skills of epidemiology and public health since John Snow’s investigation of cholera in 19th century London (20). Developing our GIS expertise has given us 21st-century tools for these key skills. All epidemiologists should have GIS in their toolbox.

Communication skills: A picture is worth a thousand words. A well-crafted map communicates patterns of disease and risk factors far more quickly and clearly than a data table can. Well-crafted maps lead to increased understanding among public health program staff members, health department leadership, and the public.

Leadership and systems thinking skills: Our work in GIS demonstrates our commitment to workforce development and to developing and enacting chronic disease epidemiology efforts that support our health agency’s mission. When hiring new epidemiologists we look for GIS skills, and we have worked to develop GIS skills in our current team of chronic disease epidemiologists. Health department leadership buy-in is needed to ensure staff time for GIS work and investment in training. Creating even a small number of basic maps on key issues can help develop that buy-in.

### New Jersey Department of Health

#### Question 1: How is your health department using GIS to address chronic disease priorities?

GIS and maps have allowed the New Jersey Department of Health (NJDOH) programs to make informed decisions on financial planning, policy development, program planning, and resource allocation regarding chronic disease. Using GIS to produce maps provides a mechanism by which public health professionals can visualize disease trends and the conditions that could be affecting trends in specific geographic areas — where we live, work, and play.

Financial planning: For program year 2019, NJDOH used maps to make funding decisions. We created maps showing disease burden, provider locations, demographic information (race, sex, education level), and causes of death. These maps have been especially helpful to the Heart Disease and Stroke Prevention Program where models were developed to assist New Jersey–based health care organizations in meeting nationally recognized best practices and standards to prevent and treat heart disease and stroke. The Office of Tobacco Control, Nutrition, and Fitness (OTCNF) used maps to identify members for the Tobacco Control Network, and to view utilization patterns for the New Jersey QuitLine. The New Jersey Cancer Education and Early Detection program and the Office of Cancer Control and Prevention (OCCP) have historically allocated state and federal funds to address the needs in all 21 New Jersey counties. However, as resources dwindle, we recognized the need to be more strategic in funding programs. Maps were instrumental in obtaining the support of leadership to reallocate and redirect funding for certain geographic regions.

Asset mapping: NJDOH uses GIS to identify assets that either impede or facilitate residents’ abilities to take advantage of opportunities for screening for breast, cervical, prostate, and colorectal cancer or to complete follow-up care. We mapped resources such as transportation, medical specialists, and other services that coincide with or are required for complete patient care. Asset mapping has also been very helpful in identifying potential partnerships at the community level. Those partnerships include faith-based organizations that were instrumental in delivering health messages to African American men, health care providers who deliver screening services, worksite wellness sites, and employer groups with at least 50 employees where screening, education, and promotion of wellness policies by the employer are needed.

Funding application development: NJDOH uses GIS to support funding applications by demonstrating the need and diversity of the state’s residents. These maps inform the development of culturally appropriate programming and services for population groups that are affected by specific health concerns. For instance, the NJDOH application for CDC’s WISEWOMAN grant proposal used maps to target specific populations and geographic locations. If funded, the maps will be instrumental in the implementation of the goals and objectives; if not funded, these maps have use across programs and will be a source of support and information for current and future projects.

#### Question 2: What are the benefits and challenges of using GIS to address the chronic disease priorities in your health department?

Benefits: Mapping chronic disease incidence, prevalence, and mortality rates, including cancer, has allowed NJDOH to develop an integrated, collaborative, and multidisciplinary plan for addressing these health concerns through strategic partnerships at the community level and within NJDOH. For example, links between cancer and obesity, poor eating habits, lack of exercise, and smoking have led to collaborative education and awareness campaigns between the OTCNF and the OCCP. The maps reflect where high prevalence of these often comorbid conditions overlap. Maps of point-of-sale audits and vendor failures have allowed community partners to see where their efforts were most needed for education and policy development. Importantly, the identification and collaboration of these partners prevented duplication of efforts.

Through the OCCP’s partnership with the New Jersey State Cancer Registry (21), all available cancer data (by cancer site, sex, race, and ethnicity) is easily accessible and available to NJDOH staff, community partners, and other stakeholders. With these data, the registry develops county-level maps of cancer incidence and mortality data for the state of New Jersey.

Challenges: Availability of GIS training has been a challenge for NJDOH staff. Recently, the Integrated Health Services Branch, Division of Community Health Services, Community Health and Wellness Unit sponsored training for 16 employees across the Agency through CEHI on the development and application of maps using GIS. The Integrated Health Services Branch leadership is committed to continuing support for future GIS training of existing staff members and making this training a desired skill for new hires.

Recommendations: Senior staff members could identify staff members for GIS training and provide scheduled time to practice and advance this skill set. Where possible, allocate funds or seek funding for this purpose. Also, use free opportunities to learn and practice GIS mapping skills, such as the Health Resources and Services Administration’s Uniform Data System mapper (22) and the Healthy City tool, which provides a community research laboratory toolkit on participatory asset mapping (23). Free online GIS training for both new and advanced users is available through the GIS Exchange (24).

#### Question 3: How is your health department using GIS to enhance the Core Competencies for Public Health?

Assessment: NJDOH and the OCCP use maps to identify needs and gaps and to monitor the health of residents. Assessment is an ongoing, continual process that must be undertaken to show effectiveness and the need for new policies and programs.

### New York State Division of Chronic Disease Prevention

#### Question 1: How is your health department using GIS to address chronic disease priorities?

Over the past 8 years, the New York State Division of Chronic Disease Prevention (DCDP) has incorporated GIS into all phases of programs to address chronic disease. In program planning, maps are used to understand how the burdens of chronic disease and key risk factors, including social determinants of health, vary across communities. GIS analysis has aided in identifying specific communities in need of focused public health action (25,26). The resulting maps enabled us to communicate effectively with decision makers and engage communities. GIS has helped to visualize community assets, including certified mammography facilities, locations offering lifestyle change programs (26,27), and retail outlets offering healthy food (28), and to identify gaps and plan local action. In program monitoring, local partners provide information about community locations where evidence-based interventions are implemented (26,28). Using GIS to analyze and display these data enables stakeholders to understand the schools, worksites, childcare centers, hospitals, corner stores, and other community locations affected by these interventions. Maps of data collected by DCDP-funded grantees demonstrate accountability to stakeholders and support performance management. In program evaluation, maps are used to assess whether policy, system, and environmental changes established by grantees occur in high-need communities and have the potential to affect a sizable population (26,28). In ongoing public health surveillance, maps of key chronic disease health and risk factor indicators based on time series data illustrate the potential impact of public health action and are used to justify and advocate for additional resources.

#### Question 2: What are the benefits and challenges of using GIS to address the chronic disease priorities in your health department?

One benefit of GIS is that maps have proven to be an effective way to communicate with many audiences about geographic disparities in chronic disease burden. Public health action is local, and many of our partners identify with a specific geographic area, be it a county, town, school district, or region. When health indicator data are displayed in maps, partners see their communities in the data reports. Maps make it easier to make the case for action at specific locations and to increase receptivity for evidence-based interventions that DCDP and our partners are promoting.

One challenge with GIS is that developing a map requires more resources than making a graph or data table. In terms of staffing resources, it has taken years to develop sufficient capacity to meet the GIS needs of the major programs within DCDP. Maintaining a staff with GIS experience has become an ongoing staff resource priority in our division. With regard to time resources, it has taken time to establish realistic expectations with partners about how long it will take for GIS projects to be completed. Partners unfamiliar with GIS often lack perspective on the skill and time involved in making maps and what appear visually to be simple changes.

A second challenge is that the effectiveness of a GIS project depends on the availability of quality, geo-referenced data. Mapping data that are inherently unreliable, come from unknown or non-validated data sources, or are collected at incongruent levels of geography often do not produce meaningful information. It has been a challenge to communicate with partners that their enthusiasm and good intentions will not compensate for the absence of reliable data. Fortunately, DCDCP’s internal and external partners have developed an understanding of the data requirements for GIS projects.

#### Question 3: How is your health department using GIS to enhance the Core Competencies for Public Health?

Financial planning and management skills: A universal challenge public health practitioners face is determining how best to allocate limited resources in a jurisdiction while most effectively addressing the health needs of the population. In New York, we have used GIS to help address this challenge and support financial planning for funding programs in which DCDP issues requests for proposals. To distribute resources that support preventive services for breast and cervical cancer in New York State, service delivery data from each contractor were subject to GIS analysis. These results helped support decision making on the total number of grantees needed to achieve statewide coverage and efficiently meet the demands for preventive cancer screening. To focus funding for community interventions that promote healthy eating, physical activity, and breastfeeding, we used GIS to identify and display the high-need communities where eligible applicants were able to propose work.

Analytical/assessment skills and community dimensions of practice skills: GIS enables our division to incorporate geographic and environmental data into community assessment processes, and to display our findings in a manner that promotes community engagement. Therefore, maps have enhanced our analytical and assessment process by adding place as a key dimension and contributed to development of Community Dimensions of Practice Skills by increasing our capacity to involve community stakeholders and effectively advocate for public health action.

## Conclusion

GIS has become a critical tool for state and local health departments and has allowed them to extend the concept of geography into many aspects of their chronic disease programs. These responses reveal the extent to which health departments are using maps and spatial analyses to 1) communicate the burden of disease; 2) inform decisions about resource allocation, policy, and priority communities for intervention efforts; 3) develop culturally competent programs; and 4) assist with program planning, monitoring, and evaluation. The continued and enhanced application of GIS to chronic disease surveillance, prevention, and treatment priorities can provide valuable benefits both to health departments and to the communities they serve.
