# Erratum, Volume 16, May 23 Release

**DOI:** 10.5888/pcd16.180674e

**Published:** 2019-06-27

The essay “Application of Geographic Information Systems to Address Chronic Disease Priorities: Experiences in State and Local Health Departments” was erroneously shown as peer reviewed. The correction was made to our website on June 11, 2019, and appears online at https://www.cdc.gov/pcd/issues/2019/18_0674.htm. We regret any confusion or inconvenience this error may have caused. 

